# Transdermal Patches for Pain Relief in Orthodontic Procedures: A Narrative Review

**DOI:** 10.7759/cureus.51669

**Published:** 2024-01-04

**Authors:** Meenakshi G Agrawal, Himanshu Kanungo, Mukesh Gupta, Kratika Mishra, Akansha Soni, Navneet Agrawal

**Affiliations:** 1 Orthodontics and Dentofacial Orthopaedics, Index Institute of Dental Sciences, Indore, IND; 2 Paedodontics, Index Institute of Dental Sciences, Indore, IND

**Keywords:** diclofenac patch, orthodontic tooth movement, pain relief, premolar extraction, separator placement, transdermal drug delivery system

## Abstract

Pain relief is an integral component of any orthodontic procedure given its high association with patient compliance and treatment adherence. A transdermal drug delivery system (TDDS) is a non-invasive method of drug delivery through the skin surface that can spread the medication throughout the dermis at a predetermined rate to produce a local or systemic effect. It might be used in place of hypodermic injections and the oral medication route. A transdermal analgesic, often known as a pain reliever patch, is an adhesive patch that contains medication to treat mild-to-severe pain. Many opioids and non-steroidal anti-inflammatory drugs are currently available as patches. TDDS offers many benefits over the conventional medication delivery method. The non-invasive transdermal route or therapy has features such as excellent bioavailability, stable medication plasma concentration, and no first-pass metabolism effect. This review aims to explore the available evidence on the use of transdermal patches for pain relief in orthodontic procedures and possibly suggest recommendations based on the findings.

## Introduction and background

Orthodontic procedures inevitably cause pain and discomfort. Pain comes first in the patient’s mind among the different variables that discourage them from starting or finishing orthodontic treatment. Contrary to other dental procedures such as extractions or subgingival scaling, the discomfort experienced during orthodontic treatment tends to worsen after appointments until it eventually reaches a plateau [1].

The application of force during orthodontic procedures causes local ischemia, the recruitment of inflammatory mediators, and alterations to the periodontal ligament, which are connected cascading processes that culminate in pain. These biochemical mediators are released and expressed over hours to days, which explains why the sense of pain increases over time. Based on factors such as age, gender, and sex, a patient’s experience of pain varies greatly from person to person [2].

Pain management is one of the key issues in the success of any orthodontic procedure. Pharmacological pain management techniques include the use of non-steroidal anti-inflammatory drugs (NSAIDs) and anesthetic gels (e.g., aspirin, ibuprofen, and diclofenac sodium gel), while mechanical pain management techniques include the use of transcutaneous nerve stimulation and medicated chewable gum [3]. Low-level laser therapy has recently become another method of pain management [4]. There are no standard guidelines for pain relief during orthodontic treatment. In this area, research and consensus remain elusive.

A transdermal patch is meant for topical application and is coated with a medicine (or a drug) to administer a particular amount of the medication (or drug) over time. The active component in a transdermal patch is intended to be released continuously over many hours to days after it has been applied to the skin. By reducing the number and size of doses required to achieve the goal of systemic medication through topical application to the intact skin surface, a transdermal drug delivery system (TDDS) improves the therapeutic value and drug safety [5].

TDDS offers many benefits over the conventional medication delivery method. The non-invasive transdermal route or therapy has features such as excellent bioavailability, stable medication plasma concentration, and no first-pass metabolism effect. Transdermal patches are just as successful as oral medication administration when it comes to drug delivery, which is a crucial component of drug administration. Many analgesics, including diclofenac, ketoprofen, and others, can be given as patches.

This review aims to evaluate information on the use of various transdermal patches for pain relief during orthodontic procedures and make recommendations for their use in patients receiving orthodontic treatment.

## Review

Transdermal patches

The Food and Drug Administration approved the first transdermal patch as a motion sickness remedy in 1979 after it was created in the 1970s [5]. Transdermal patches have several advantages over oral delivery. Some of the advantages include the ability to cease drug administration by simply removing the patch, the avoidance of first-pass metabolism, sustained and non-rapid absorption, stable plasma levels over extended periods, the absence of drug reliance, the prevention of gastrointestinal distress, and the absence of drug dependence.

Components of Transdermal Drug Delivery System

TDDS include liners that shield patches from damage while they are being stored; adhesives that are used to attach the patch to the skin as well as to hold the components of the patch together; membranes that regulate the medication release from multi-layered patches; and drugs with direct contact between drug reservoir and release liner and backing which shields the patch from the surroundings.

Types of Transdermal Patches

The various types of transdermal patches in vogue are single-layer drug-in-adhesive patches, multi-layer drug-in-adhesive patches, reservoir-type patches, matrix-type patches, and vapor patches. In single-layer drug-in adhesive patches, the medicine is in contact with the adhesive layer that is bonded to the skin. Drug release is assisted by the coating of adhesive, which also clings to the skin’s various layers. in multi-layer drug-in-adhesive patches, the single-layer drug-in adhesive, which includes introducing the drug directly into the adhesive layer, is analogous to the multi-layer drug-in adhesive. One of the layers in this arrangement immediately releases the medication from the reservoir. Both a permanent backing and a temporary liner layer are included with this patch. The reservoir transdermal system, as opposed to the single-layer and multi-layer drug-in-adhesive systems, has a separate drug layer. In matrix-type patches, the system has a compartment for fluids containing a medicine solution or suspension that is kept apart from the liner by an adhesive and membrane. The backing layer also supports this patch scheme. In other patches, the adhesive layer functions as both a fume-release layer as well as a means of holding the various layers together. These patches are widely used to release essential oils for up to six hours [6].

Ideal Properties of Transdermal Drug Delivery System

An ideal TDDS should have a shelf life of up to two years; a small size (less than 40 cm^2^); a convenient dosing schedule (once daily to once weekly); should be cosmetically pleasing (clear, white hue); have simple packaging (i.e., fewer steps and pouches needed to apply the system); easy release liner removal (i.e., for young or elderly patients); and appropriate skin adhesion (i.e., simple removal without causing skin harm and no falloff during the dosage interval and no cold flow, or leftover material, around the patch’s edge during storage, after application, or after removal) [7].

Mechanism of Action of Transdermal Patches

A transdermal patch holds a medication until it is applied, serving as a carrier for it. The adhesive in the patch adheres to the skin at the site of application, allowing the medications to start seeping into the bloodstream. Different mechanisms govern how the transdermal patch works and how the active therapeutic ingredient travels from the patch via the skin to the circulatory system. A systemically active medicine must possess certain physicochemical qualities that facilitate the drug’s absorption through the epidermis and entry into the microcirculation to reach a target tissue [8].

Modern Techniques of Transdermal Drug Delivery System

Dental treatments are often marred by postoperative pain and discomfort and the analgesics prescribed may lead to undesirable side effects. The use of new and improved modern techniques of drug delivery by transdermal patches may aid in reducing the dose of drug administered without reducing efficacy.

In contrast to the topical application of medications to the skin, external stimuli, such as electrical, mechanical, or physical stimuli, are known to increase the permeability of pharmaceuticals and biomolecules through the skin. Active transdermal delivery, which is known to transport medications into the skin swiftly and reliably, is used to describe TDDS when it is complemented by the proper tools. Additionally, the therapeutic effectiveness of medications that are administered can be accelerated using this method of enhanced TDDS [9]. Examples of active delivery systems are described below [9].

Iontophoresis: Iontophoresis has been found to improve skin penetration and increase the release rate of various drugs with poor absorption/permeation profiles by encouraging ion migration across the membrane under the influence of a small externally applied potential difference (less than 0.5 mA/cm^2^).

Sonophoresis: Transdermal medication administration can be improved using an ultrasonic device that produces the necessary range of ultrasound frequencies. Because it enhances drug circulation by establishing an aqueous channel in the disturbed bilayer by cavitation, low-frequency ultrasound is more efficient.

Electroporation: This method includes applying high-voltage electric pulses to the skin over short periods (times), resulting in the formation of small holes in the SC that increase permeability and facilitate drug transfer.

Photomechanical waves: The SC can be penetrated by photodynamic waves that are directed at the skin, allowing the medication to enter via the momentarily formed channel. Low radiation exposure of about 5-7 J/cm^2^ is used to increase the depth to 50-400 m for successful transmission. The incident wave induces limited ablation.

Microneedle: This technique uses micron-sized needles to puncture the epidermal layer of the skin to cause medication diffusion. These microneedles transport medications straight to the blood capillary area for active absorption because they are short and thin, which aids in reducing pain.

Thermal ablation: Thermophoresis, also known as thermal ablation, is a promising method for creating microchannels in the skin that increase medication delivery by selectively breaking the stratum corneum structure with localized heat.

Chemical penetration enhancers that have been shown to have superior skin penetration to those of individual chemicals can be employed alone or in combination with penetration enhancers. Eutectic mixtures and nanoparticle composite self-assembled vesicles are examples of these synergistic systems.

Vesicles: Vesicles are colloidal particles filled with water composed of bilayers of amphiphilic molecules. These amphiphilic compounds create multilayer vesicles, which are concentric bilayers with one or more shells when there is an excess of water present. Fat- and water-soluble drugs can be transported via vesicles for transdermal absorption. Vesicles can be used to create a continuous release of medicines when applied topically.

Polymeric nanoparticles: According to their composition, nanoparticles, which are nanocarriers with diameters ranging from 1 to 1,000 nm, can be divided into several different categories. When a drug is administered in the form of nanoparticles, it exhibits a targeted and controlled release pattern, changes in the drug’s in vivo dynamics, and extends its stay in the blood, all of which improve drug bioavailability and reduce toxicity and adverse effects.

Nanoemulsion: Low viscosity, isotropy, thermodynamic stability, and dynamic stability are the characteristics of a nanoemulsion. Nanoemulsions have exceptional wettability that guarantees close contact with the skin due to their small particle size, large specific surface area, and low surface tension. Numerous additional advantages of nanoemulsions include their great solubilization capacity, physical stability, enhanced bioavailability, simplicity of manufacture, low energy requirement, and long shelf life.

Characterization Techniques for Transdermal Drug Delivery System

In TDDS, evaluating delivery efficacy and efficiency is crucial. Depending on the approach and Jeong et al., there are many different methods used for this. However, the three most used techniques employ tape stripping, diffusion cells, and microscopic and spectroscopic inspection. All of these characterization techniques are based on the idea of measuring the amount of the drug in each surface layer as it is absorbed or of storing an imaging material in place of the drug to visually check the absorption pattern [10,11].

A cross-over efficacy trial was conducted by Bhaskar et al. in 2010 [12]. They compared transdermal patches with oral medication (both containing diclofenac) for postoperative analgesia after multiple orthodontic premolar extractions and concluded that TDDS is superior to oral diclofenac. Due to the once-daily application and less frequent occurrence of systemic adverse effects, respondents claimed that they felt more comfortable using the transdermal patch. The study’s findings suggested that the transdermal diclofenac patch can be used for routine post-extraction analgesia and offers just as effective analgesia as oral diclofenac tablets, with the added benefit of higher patient compliance [12].

Krishnan et al. [13] (2015) investigated the efficacy of diclofenac transdermally and orally in post-extraction pain management. The 40 patients were divided into six groups, each with 40 unsalvageable non-tender molars. The visual analog scale (VAS) was used to assess postoperative pain at six and 12 hours. Patches containing diclofenac did not outperform diclofenac sodium oral tablets in terms of efficacy in controlling postoperative molar extraction pain [13].

Kumar et al. [14] performed a similar study in 2017 in which they chose 100 adults who had undergone major dental surgery. The transdermal diclofenac patch was placed 2 to 3 cm away from the surgical site. The other NSAIDs were stopped and the patch was replaced every 24 hours, and a scale from 1 to 5 was used to record the level of pain. All data was gathered, and statistical analysis (the chi-square test) was performed. The degree of pain relief varied from excellent in 34% to good in 38%, fair in 27%, and poor in only 1% of the patients after the application of an analgesic transdermal patch [14].

A recent study conducted by Talnia et al. [15] (2020) evaluated the efficacy of a transdermal diclofenac patch versus an oral diclofenac tablet as an analgesic following premolar extractions in orthodontic patients. The study’s findings indicated that transdermal diclofenac patches were marginally more effective than oral diclofenac tablets in reducing postoperative pain following extractions performed for orthodontic purposes, but statistical analysis using the chi-square test found no statistically significant difference (p > 0.05). When used to alleviate premolar orthodontic extraction pain of mild-to-moderate severity, a transdermal diclofenac patch demonstrated potential as an analgesic method with a lower incidence of systemic side effects. They also concluded that the use of transdermal patches may be constrained by price and accessibility [15].

George et al. [16] (2021) studied the analgesic efficacy of dermatological patches in 20 orthodontic patients who had begun receiving orthodontic treatment. Out of these, 10 patients received transdermal diclofenac 200 mg patches, and each patient received a proforma with a pain chart and a table showing the relationship between time and discomfort. The other 10 patients received a proforma without a transdermal patch. The patient noted their level of pain at intervals of one hour, four hours, 12 hours, one day, and two days. The patient’s proforma was then collected after two days. Following that, all data were calculated in Microsoft Excel, statistical analyses were performed using SPSS software, and the outcomes were analyzed. It was noted that the patients began to experience pain at the end of an hour. The transdermal patch’s activity followed afterward. In the patient wearing the transdermal patch, the pain marginally decreased after four hours. After 12 hours, the intensity in practically every category was the same, with a 1% difference. However, after a single day, the patients using transdermal patches had a significant edge over the patients who were not wearing them. There was a statistically significant difference between them of up to 1.5%. The patient-’s reported level of pain at the end of two days provided the study’s final measurement. According to the study’s findings, transdermal analgesic patches have a significant edge when it comes to providing pain relief during dental procedures. Consequently, transdermal analgesic patches may be used as an analgesic in place of oral analgesics [16].

Khadase et al. [17] (2022) conducted another recent randomized controlled trial among 30 patients comparing oral diclofenac transdermal patch to oral diclofenac sustained-release tablet as a postoperative analgesic after orthodontic extractions of premolar teeth. The five-point Verbal Pain Intensity and Pain Alleviation Score Charts were used to assess pain alleviation and intensity with both diclofenac formulations. They concluded that using oral diclofenac pills and the transdermal patch resulted in a progressive increase in pain relief scores and a steady decrease in pain intensity scores [17].

Shankar et al. [18] (2021) compared the efficacy of a transdermal diclofenac patch and a ketoprofen patch as postoperative analgesia after the extraction of first premolars bilaterally in both arches for orthodontic purposes in 52 patients between the ages of 15 and 25 years. The first premolars in the mandible and maxilla were extracted bilaterally for orthodontic purposes using the split-mouth approach. Following premolar extraction from the first and fourth quadrants, one ketoprofen patch was applied, whereas a diclofenac patch was used following premolar extraction from the second and third quadrants. All extractions were performed under the influence of local anesthesia. Using the Student’s t-test, the data were compiled and statistically examined. Diclofenac and ketoprofen patches had mean VAS scores of 2.05 (0.75) and 1.09 (0.3), respectively. Overall, 13 (25%) patients and one (1.9%) patient using diclofenac and ketoprofen patches, respectively, needed further medicine. No significant complications or negative outcomes were noticed in any of the groups. After orthodontic extraction, transdermal patches containing diclofenac or ketoprofen were both effective in reducing discomfort. In comparison to patients who received a ketoprofen patch, those who received a diclofenac patch needed greater extra analgesia within 24 hours. All medications were well tolerated by the patients and exhibited no notable side effects. The authors concluded that more research with a larger sample size is required to assess the effectiveness of transdermal patches in diverse surgical procedures [18].

Another recent study conducted by Pandey et al. [19] (2023) compared the analgesic efficacy of diclofenac 200 mg and ketoprofen 30 mg transdermal patches for post-orthodontic exodontia pain. The study comprised 30 patients who underwent local anesthetic for the extraction of bilateral maxillary and/or mandibular premolars during orthodontic treatment. In the two sessions, which were scheduled in random order, each patient received one transdermal diclofenac 200 mg patch and one transdermal ketoprofen 30 mg patch on the outside, ipsilateral upper arm immediately following the extraction. Using VAS, the pain score was collected every two hours during the first 24 hours following surgery. It was documented how often rescue analgesics were needed and how many rescue analgesics were used in the first 24 hours following surgery. Additionally, any transdermal patch allergy symptoms were noted. Using the Mann-Whitney U test, the analgesic efficacy of the two transdermal patches at any given point in the 24-hour period did not differ statistically significantly (p = 0.05). By comparing VAS pain scores at various time points to those at zero to two hours following the application of transdermal ketoprofen and diclofenac patches, respectively, Wilcoxon matched-pair test results showed an overall intragroup statistically significant difference (p = 0.05). The transdermal patch of ketoprofen (2.33) had a somewhat lower mean maximum pain intensity than the transdermal patch of diclofenac (2.60). Within the first 12 hours following surgery, patients used rescue analgesics, with the average number of rescue analgesics taken being slightly lower with ketoprofen transdermal patch treatment (0.23 vs. 0.27). They concluded that the analgesic effects of ketoprofen and diclofenac transdermal patches were comparable after orthodontic extraction. The first few hours of the postoperative follow-up period were the only time patients needed rescue analgesics [19].

We conducted an indigenous trial at the Index Institute of Dental Sciences, Indore, India, to compare the clinical effectiveness of analgesia by transdermal diclofenac patches and with herbal patches following multiple elastic separator placement in patients undergoing orthodontic treatment. In this study, elastic separator placement was done after obtaining patient consent in a standardized manner and then analyzed using statistical analysis of data derived from VAS scoring done by patients. Patients with full complimentary teeth except third molars with healthy periodontal status, gingival health, and good oral hygiene were included in the study while those with a history or clinical evidence of allergic and contraindication to NSAIDs were excluded from the study. Patients were divided into three groups of 22 patients each. In group A, diclofenac di-ethylamine 200 mg transdermal patches were given. In group B, herbal transdermal patches were given. Group C was the control group where placebo patches were given. All patients were instructed to take ketorolac 10 mg tablet on an SOS basis. The pain intensity was assessed using a modified VAS after four, eight, 12, 24, 48, and 72 hours. The pain intensity as reported by the participants showed a statistical difference in pain relief between the transdermal patch groups and the placebo group (p < 0.05). There was no significant difference between the two groups at four, eight, 12, 24, and 48 hours (p > 0.05). The study concluded that transdermal patches are an effective modality for pain relief following elastic separator placement and both transdermal diclofenac patches and transdermal herbal patches can be used for pain control.

Table 1 summarizes the available literature on the use of TDDS in orthodontic treatment.

**Table 1:** Studies on the use of transdermal patches for pain relief in orthodontic procedures. TDDS: transdermal drug delivery system; VAS: visual analog scale

Year of publication	Authors	Type of study	Study protocol	Number of participants	Result
2010	Bhaskar et al. [12]	Cross-over efficacy trial	Comparison of TDDS diclofenac patch (100 mg) vs. oral diclofenac (50 mg) following multiple premolar extraction	22	Transdermal diclofenac patches offer equally powerful analgesia as oral diclofenac tablets with the added advantage of higher patient compliance
2015	Krishnan et al. [13]	Randomized controlled double-blind study	Patients undergoing dental extractions were divided into two groups, with one group (n = 20) receiving a placebo patch and an orally active medication of diclofenac sodium (75 mg twice daily dosage) while the other group (n = 20) was provided with a diclofenac patch (200 mg) and an oral placebo tablets	40	TDDS is comparable to oral diclofenac in pain relief following dental extraction procedures
2020	Talnia et al. [15]	Quasi-experimental study	Comparison of a transdermal diclofenac patch (100 mg) versus an oral diclofenac tablet (50 mg twice a day) as analgesic following premolar extractions in orthodontic patients	33	With a lower incidence of systemic side effects, the transdermal diclofenac patch demonstrated potential as an analgesic modality for the management of mild-to-moderate intensity pain during premolar orthodontic extraction
2021	George et al. [16]	Quasi-experimental study	20 were selected randomly after the placement of the initial NiTi wire in mild crowding 0.1–4 mm little irregularity index without extraction of teeth and were given either a transdermal patch 100 mg (n = 10) or no analgesic (n = 10). Pain intensity was noted after two days using a pain chart	20	TDDS had a significant analgesic effect, with its use being recommended in routine dental practice
2021	Shankar et al. [18]	Randomized controlled study	Comparison of the efficacy of a transdermal diclofenac patch (200 mg) with a ketoprofen patch (20 mg) as postoperative analgesia after extraction of the first premolars bilaterally in both arches for orthodontic purposes	52	Compared to patients who received a ketoprofen patch, those who received a diclofenac patch needed greater extra analgesia within 24 hours
2022	Khadase et al. [17]	Quasi-experimental study	Comparison of TDDS diclofenac patch (100 mg) vs. oral diclofenac sustained released (100 mg) following orthodontic premolar extractions	30	TDDS is comparable to oral diclofenac in pain relief following orthodontic premolar extractions
2023	Pandey et al. [19]	Randomized split-mouth study	Patients undergoing orthodontic bilateral maxillary and/or mandibular premolar extractions received a single transdermal diclofenac 200 mg patch and a single transdermal ketoprofen 30 mg patch on the outer, ipsilateral upper arm immediately post-extraction in the two appointments in random order. Pain was recorded using the VAS scale after 24 hours	30	Similar analgesia is provided following orthodontic extraction by ketoprofen and diclofenac transdermal patches

## Conclusions

Given the evidence of its established analgesic effectiveness with a decreased incidence of systemic adverse effects, transdermal patches appear to be a potential analgesic modality for the management of mild-to-moderate pain in orthodontic procedures. Transdermal therapy, possibly with a stronger analgesic in the transdermal patch, may also be useful in treating post-extraction and post-insertion pain in routine orthodontic procedures. Future prospects of transdermal use include the use of fentanyl, buprenorphine, and morphine patches to relieve cancer pain; transdermal estrogen in metastatic prostate cancer; granisetron; and other antiemetic medications to help prevent nausea and vomiting associated with chemotherapy. Research on these and other related topics is either presently being conducted or has already been completed. The only limitation of transdermal patches includes their propensity to irritate the skin due to the adhesive or the drug component. Hence, we conclude that transdermal analgesic patches must be considered as the preferred choice for effective pain management in orthodontic procedures. However, the true scope of transdermal patches can only be determined if more robust randomized controlled trials with larger patient populations are conducted.

## References

[1] Pollat O (2007). Pain and discomfort after orthodontic treatment. Semin Orthod.

[2] Abu Alhaija ES, Aldaikki A, Al-Omairi MK, Al-Khateeb SN (2010). The relationship between personality traits, pain perception and attitude toward orthodontic treatment. Angle Orthod.

[3] Roth PM, Thrash WJ (1986). Effect of transcutaneous electrical nerve stimulation for controlling pain associated with orthodontic tooth movement. Am J Orthod Dentofacial Orthop.

[4] Lim HM, Lew KK, Tay DK (1995). A clinical investigation of the efficacy of low level laser therapy in reducing orthodontic postadjustment pain. Am J Orthod Dentofacial Orthop.

[5] Bird D, Ravindra NM (2020). Transdermal drug delivery and patches—an overview. Med Devices Sens.

[6] Patel DS, Patel MV, Patel BA, Patel PA (2012). Transdermal patches: a complete review on transdermal drug delivery system. Int J Pharm Res Sch.

[7] Alkilani AZ, Nasereddin J, Hamed R, Nimrawi S, Hussein G, Abo-Zour H, Donnelly RF (2022). Beneath the skin: a review of current trends and future prospects of transdermal drug delivery systems. Pharmaceutics.

[8] Rasheed SH, Haribabu R, Mohiddin Md. K (2011). Transdermal drug delivery system. Simplified medication regimen- a review. Res J Pharm Bio Chem Sci.

[9] Jeong WY, Kwon M, Choi HE, Kim KS (2021). Recent advances in transdermal drug delivery systems: a review. Biomater Res.

[10] Raza R, Mittai A, Kumar P (2015). Approaches and evaluation of transdermal drug delivery system. Int J Drug Dev Res.

[11] Sheth NS, Mistry RB (2011). Formulation and evaluation of transdermal patches and to study permeation enhancement effect of eugenol. J Appl Pharm Sci.

[12] Bhaskar H, Kapoor P, Ragini Ragini (2010). Comparison of transdermal diclofenac patch with oral diclofenac as an analgesic modality following multiple premolar extractions in orthodontic patients: a cross over efficacy trial. Contemp Clin Dent.

[13] Krishnan S, Sharma P, Sharma R, Kumar S, Verma M, Chaudhary Z (2015). Transdermal diclofenac patches for control of post-extraction pain. Pilot randomized controlled double-blind study. Oral Maxillofac Surg.

[14] Kumar V, Gupta S, Verma R (2017). Evaluation of the role of transdermal diclofenac patch (Nupatch) in management of pain in postperative patients. Int J Contemp Med Res.

[15] Talnia S, Fry RR, Sharma A, Patidar DC, Goyal S, Gandhi G (2020). Efficacy of transdermal diclofenac patch as an analgesic following premolar extractions in orthodontic patients. Ann Maxillofac Surg.

[16] George A, Kumar N, Ganapathy D (2021). Analgesic effect of dermatological patch in orthodontic patients. Eur J Mol Clin Med.

[17] Khadase P, Goyal R, Nirangjan AP, Vaidehi A, Chakraborty AJ, Kishor A (2022). Comparison of oral diclofenac transdermal patch versus oral diclofenac sustained release tablet as a post-operative analgesia following orthodontic extractions of premolar teeth: a randomized clinical study. Int J Health Sci.

[18] Shankar D, Sinha A, Anand S, Verma N, Choudhary S (2021). Efficacy of transdermal diclofenac patch and ketoprofen patch as postoperative analgesia after extraction of first premolars bilaterally in both arches for orthodontic purpose: a comparative study. J Pharm Bioallied Sci.

[19] Pandey K, Shettar V, Kale T (2023). Efficacy of transdermal ketoprofen patch in comparison to transdermal diclofenac patch in postoperative analgesia for orthodontic extractions: a randomized split-mouth study. Cureus.

